# First Principle Study of TiB_2_ (0001)/γ-Fe (111) Interfacial Strength and Heterogeneous Nucleation

**DOI:** 10.3390/ma14061573

**Published:** 2021-03-23

**Authors:** Qin Wang, Peikang Bai, Zhanyong Zhao

**Affiliations:** School of Materials Science and Engineering, North University of China, Taiyuan 030051, China; wq950724@163.com (Q.W.); baipeikang@nuc.edu.cn (P.B.)

**Keywords:** laser processing, metals and alloys, first principle, interfacial strength, heterogeneous nucleation

## Abstract

TiB_2_/316L stainless steel composites were prepared by selective laser melting (SLM), and the adhesion work, interface energy and electronic structure of TiB_2_/γ-Fe interface in TiB_2_/316L stainless steel composites were investigated to explore the heterogeneous nucleation potential of γ-Fe grains on TiB_2_ particles using first principles. Six interface models composed of three different stacking positions and two different terminations were established. The B-terminated-top 2 site interface (“B-top 2”) was the most stable because of the largest adhesion work, smallest interfacial distances, and smallest interfacial energy. The difference charge density and partial density of states indicated that a large number of strong Fe-B covalent bonds were formed near the “B-top 2” interface, which increased the stability of interface. Fracture analysis revealed that the bonding strength of the “B-top 2” interface was higher than that of the Fe matrix, and it was difficult to fracture at the interface. The interface energy at the Ti-poor position in the “B-top 2” interface model was smaller than that of the γ-Fe/Fe melt, indicating that TiB_2_ had strong heterogeneous nucleation potency for γ-Fe.

## 1. Introduction

316L stainless steel is widely used in aerospace, biomedicine, automotive structure, and other fields due to its high wear resistance, corrosion resistance, and ductility [1]. However, it shows poor mechanical properties under high strength and high temperature, limiting its application in the industry [2,3]. Metal matrix composites have received great attention in many applications [4]. The mechanical properties of stainless-steel matrix composites, such as strength, hardness, wear resistance, and thermodynamics, can be effectively improved by adding ceramic reinforcement phase [1,5]. The commonly used ceramic reinforcing phases are TiC, TiB, TiB_2_, SiC, WC, etc [6,7,8,9,10,11]. Because the TiB_2_ particle has the advantages of good thermal stability, high hardness, and good compatibility with steel. It is an effective grain refiner and an ideal material for strengthening steel matrix composites [12,13,14].

Many experiments have shown the effect of TiB_2_ on the microstructure and mechanical properties of stainless steel. AlMangour et al. [2] found that TiB_2_/316L stainless steel composite formed by selective laser melting (SLM) has higher yield strength, hardness, and wear resistance, the hardness can be increased by about twice with 15 vol% TiB_2_. Liu et al. [15] prepared in-situ synthesized TiB_2_/Mg-based composites by powder and explored the grain refining effect of the TiB_2_ phase. They found that TiB_2_ particles can be used as heterogeneous nucleation centers of magnesium alloys, effectively refining α-Mg grains. 

In any kind of composite, the interfacial reaction and bonding between the matrix and reinforcement play a major role, which determines the effective heterogeneous nucleation ability and quality of the composites. However, due to the complexity of interface structure, it is difficult to evaluate the detailed interaction of the ceramic phases and matrix interface on the atomic or electronic scale through experimental methods. To further explore the strengthening mechanism and refinement mechanism of ceramic particles on the substrate metal, many scholars use the first principle to calculate and analyze the interface bonding. Liu et al. [16] studied the interface structure of Mg (0001)/TiB_2_ (0001) by using the first principles and analyzed the heterogeneous nucleation potential of α-Mg on TiB_2_. The results showed that the interface energy of Mg/TiB_2_ was greater than that of α-Mg/Mg melt, which was not conducive to heterogeneous nucleation. Deng et al. [17] revealed the grain refinement mechanism of Al-Ti-B in Al-Si alloy by analyzing the Al/TiB_2_ interface energy, indicating that a new phase Al_3_Ti was formed at the Ti-rich termination, which could be used as a nucleation center. Bai et al. [18] prepared TiC/316L stainless steel by SLM and calculated the interface properties of TiC/γ-Fe using first principles, indicating that TiC can promote the heterogeneous nucleation of γ-Fe. It has been common to explore the heterogeneous nucleation potential and grain refinement effects of TiB_2_ in 316L stainless steel composites through experiments [13,19], unfortunately, the atomic interaction at the interface level is currently not entirely clear. Thus, it is highly desirable to investigate the interfacial stability of TiB_2_/316L composites based on first-principles calculation. 

In this paper, SLM was used to fabricated TiB_2_/316L stainless steel composites. The bulk properties, surface behavior, adhesion work, interface energy, electronic properties of the TiB_2_ (0001)/γ-Fe (111) were explored by first-principles calculation to reveal interfacial strength and heterogeneous nucleation potential of γ-Fe on TiB_2_ atoms. The bulk properties test is to make the simulated value closer to the experimental value and ensure the accuracy of calculation. The purpose of surface energy test is to get a stable surface model and lay a foundation for the interface calculation. Interfacial adhesion and bonding nature play an important role in the theoretical research of nucleation potency of a heterogeneous substrate. The larger the adhesion work is, the smaller the interface distance is, the smaller the interface energy is, and the more stable the interface structure is, when the interfacial energy between TiB_2_ and γ-Fe is lower than that between γ-Fe and Fe melt, which is beneficial to effective heterogeneous nucleation.

## 2. Experimental and Computational Procedures

**Figure 1:**
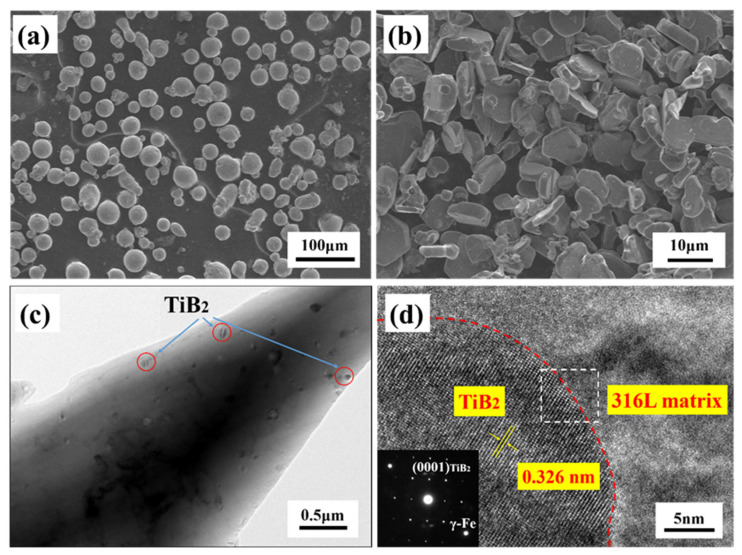
(**a**) The SEM morphology of 316L stainless steel powder; (**b**) The SEM morphology of TiB_2_ powder; (**c**) Bright-field TEM image of distribution of TiB_2_ particles in the 316L matrix grain; (**d**) HRTEM and corresponding FFT patterns of the TiB_2_/316L stainless steel composites.

The 316L stainless steel powder (particle size of 15–45 μm) prepared by gas atomization was used for this experiment, Figure 1a. TiB_2_ with the particle size of 5–10 μm was used as reinforcement phase, Figure 1b. TiB_2_/316L stainless steel composite was fabricated by using Renishaw AM 250 with a 220 W laser power, a 900 mm/s scanning speed, a 100 μm laser beam diameter, and an 80 μm hatch spacing. The whole printing process was carried out under the protection of argon. The transmission electron microscopy (JEM-2100F, Tokyo, Japan) was used to observe the microstructure and orientation relationship. 

All calculations used in this paper were performed in the Cambridge Serial Total Energy Package (CASTEP, Material Studio) based on density functional theory [20,21]. The exchange-correlation select generalized gradient approximation (GGA) with the Perdew–Burke–Ernzerhof (PBE) [22]. The interaction between ions and covalent electrons was described by the ultrasoft pseudopotential method. Broyden Fletcher Goldfarb Shanno algorithm (BFGS) was used in geometry optimization to minimize the ground state and energy of the atom [23]. The value of plane-wave cutoff energy was set to 410 eV, k point was set to 10 × 10 × 1. The convergence criteria are as follows: 2 × 10^−5^ eV/atom for the energy, 0.1 GPa for maximum stress, 0.05 eV/Å for maximum force, and 0.003 Å for maximum displacements.

## 3. Results and Discussion

### 3.1. Experiment

Due to the small amount of TiB_2_, which is only 0.3%, it is difficult to observe under optical microscope and scanning electron microscope. Only under TEM can nano-sized TiB_2_ particles be found. Figure 1c shows the TEM of TiB_2_/316L matrix composites. It can be seen from Figure 1c that most of the TiB_2_ particles are distributed in the 316L matrix grains. The HRTEM morphology and corresponding FFT patterns of TiB_2_ and 316L matrix interface are shown in Figure 1d. The interplanar spacing of the TiB_2_(0001) planes was measured to be 0.326 nm. The main phase component in 316L stainless steel is γ-Fe, other phase components were ignored, and in the low index surface of γ-Fe, the mismatch between γ-Fe (111) and TiB_2_ (0001) is less than 15%, which belongs to the semi-coherent interface structure, and the interface bonding is good. The mismatches between TiB_2_ (0001) and other planes of γ-Fe are greater than 15%, which is not conducive to the formation of a stable interface. To explore the interfacial bonding strength and interaction of TiB_2_ reinforced 316L stainless steel composite, we investigated the interfacial stability of TiB_2_(0001) and γ-Fe(111) matrix using first principle calculations as follows.

### 3.2. Calculation and Simulation

#### 3.2.1. Bulk and Surface Properties

- Bulk Properties

According to the simulation parameters in the second section, the geometric optimizations of γ-Fe (111) and TiB_2_ (0001) were carried out. The lattice constants, volume, bulk modulus, and formation enthalpy of γ-Fe and TiB_2_ are listed in Table 1. The optimized lattice constants of γ-Fe and TiB_2_ obtained in this calculation are basically consistent with the experimental and other calculated values, which ensure that the parameter setting is reliable and accurate.

- Surface Energy

The convergence test method was used to obtain the optimal atomic layers before calculating the surface energy. For γ-Fe (111) surface model, the surface energy can be expressed as follows [28]:

(1) $$\sigma \;=\frac{E_{\mathrm{Slab}}^{N}-N\Delta E}{2A}$$

where $E_{\mathrm{Slab}}^{N}$, is the total energy of an N-layer slab, ΔE is the incremental energy obtained by $(E_{\mathrm{Slab}}^{N}-E_{\mathrm{Slab}}^{N-2})/2$, A is the surface area, N is the number of atom layers of the surface model. In order to eliminate the periodic effect between the surface atoms, a vacuum layer of 10 Å was added on each surface when the surface model was constructed. The surface energies of γ-Fe (111) models with different atomic layers are listed in Table 2. The results show that the surface can converge to about 2.56 J/m^2^ when the number of atomic layers reaches seven, which satisfies the requirement of a stable state. In this case, γ-Fe has also achieved characteristics of bulk phase. The results are well in line with Shi et al. [29].

There is only one kind of atom in the termination of the TiB_2_ surface, namely Ti-termination or B-termination. The three, five, seven, nine, and eleven atomic layers were selected for the convergence test to eliminate the pseudo dipole effect on the surface of TiB_2_. The change of interlayer distances of TiB_2_(0001) surfaces with different termination atoms after relaxation is shown in Table 3.

With the increase of the number of atomic layers, the interlayer distance of atoms decreases, and the atoms show characteristics of relaxation from the seventh layer. Therefore, the surface structure of Ti and B termination can be similar to characteristics of bulk phase when the layer thickness n ≥ 9. Therefore, the number of atomic layers of TiB_2_ (0001) is selected as nine.

TiB_2_ is a multi-element phase and exhibits a non-stoichiometric, whose surface energy is closely related to the chemical potential of different termination atoms. The surface energies of TiB_2_ (0001) are calculated [30]:

(2) $$E_{\mathrm{TiB}2}=\frac{1}{2A}{[E}_{\mathrm{slab}}-N_{B}\mu_{B}-N_{\mathrm{Ti}}\mu_{\mathrm{Ti}}+\mathrm{PV}-\mathrm{TS}]$$

where $E_{\mathrm{Slab}}^{}$ is the total energy after fully relaxed surface, $N_{\mathrm{Ti}}$ and $N_{B}$ represent the numbers of Ti and B atoms in the slab, $\mu_{\mathrm{Ti}}$ and $\mu_{B}$ slab are the chemical potentials of Ti and B atoms, respectively, A stands for the surface area. The surface energy of TiB_2_ was calculated in the ideal state of CASTEP module, so the simulation is carried out at 0 K, the PV and TS terms in the formula can be ignored. The formula is simplified to:

(3) $$E_{{\mathrm{TiB}}_{2}}=\frac{1}{2A}{[E}_{\mathrm{slab}}-N_{B}\mu_{B}-N_{\mathrm{Ti}}\mu_{\mathrm{Ti}}]$$

(4) $$\mu_{\mathrm{Ti}}^{\mathrm{bulk}}{+2\mu }_{B}^{\mathrm{bulk}}{+\Delta H}_{f}^{0}{(\mathrm{TiB}}_{2}{)=\mu }_{\mathrm{Ti}}{+2\mu }_{B}{=\mu }_{{\mathrm{TiB}}_{2}}^{\mathrm{bulk}}$$

According to the above formula, Equation (2) can be expressed as

(5) $$E_{{\mathrm{TiB}}_{2}}=\frac{1}{2A}{[E}_{\mathrm{slab}}-\frac{1}{2}N_{B}\mu_{{\mathrm{TiB}}_{2}}^{\mathrm{bulk}}+\;(\frac{1}{2}N_{B}-N_{\mathrm{Ti}}{)\mu }_{\mathrm{Ti}}]$$

The chemical potential of Ti or B element in the bulk should be larger than that of the corresponding surface slab:

(6) $$\mu_{\mathrm{Ti}}^{}\leq \mu_{\mathrm{Ti}}^{\mathrm{bulk}}{,\mu }_{B}^{}\leq \mu_{B}^{\mathrm{bulk}}$$

Combining Equations (4) and (6), the formation enthalpy (${\Delta H}_{f}^{0}{(\mathrm{TiB}}_{2})$) can be defined:

(7) $${\Delta H}_{f}^{0}{(\mathrm{TiB}}_{2})\leq \mu_{\mathrm{Ti}}-\mu_{\mathrm{Ti}}^{\mathrm{bulk}}\leq 0$$

(8) $${\Delta \mu }_{\mathrm{Ti}}{=\mu }_{\mathrm{Ti}}-\mu_{\mathrm{Ti}}^{\mathrm{bulk}}$$

**Figure 2:**
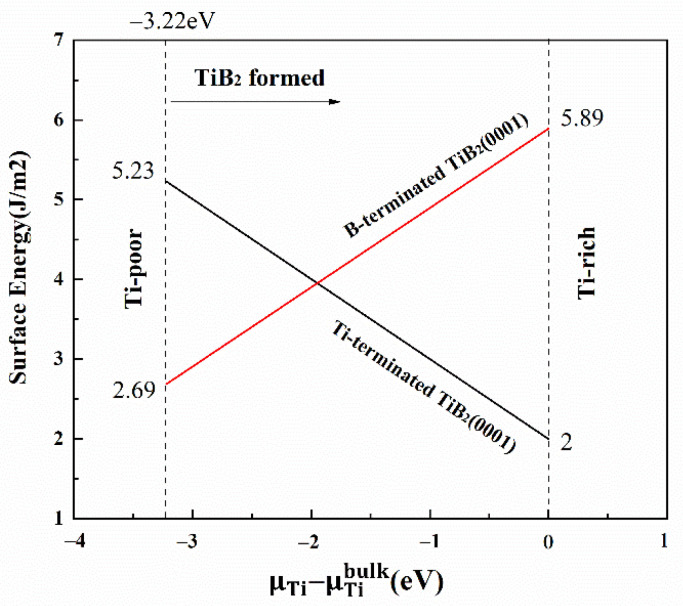
The relation between TiB_2_ (0001) surface energy of the difference of titanium chemical potential.

The surface energies of TiB_2_ (0001) with B and Ti termination are shown in Figure 2. The calculated value ${\Delta H}_{f}^{0}{(\mathrm{TiB}}_{2})$ is −3.22 eV. The calculated values of the surface energy of B and Ti terminated of TiB_2_(0001) surface are in the ranges of 2.69 to 5.89 J/m^2^ and 5.23 to 2 J/m^2^, respectively, which are consistent with the results reported in Ref [17]. With the increase of the ${\Delta \mu }_{\mathrm{Ti}}$, the surface energy of B-termination decreases linearly, while that of Ti-termination is the opposite. A more stable interface structure has smaller surface energy. When ${\Delta \mu }_{\mathrm{Ti}}$ is less than −1.85eV, the surface energy of B-termination is smaller than that of Ti, thus, the structure of B-termination is more stable. On the contrary, the surface energy of Ti-termination is smaller in the range of −1.85 to 0 eV, which indicates that the Ti-termination model is more stable.

#### 3.2.2. Properties of the TiB_2_/γ-Fe Interface

- TiB_2_ (0001) and γ-Fe (111) Interface

**Figure 3:**
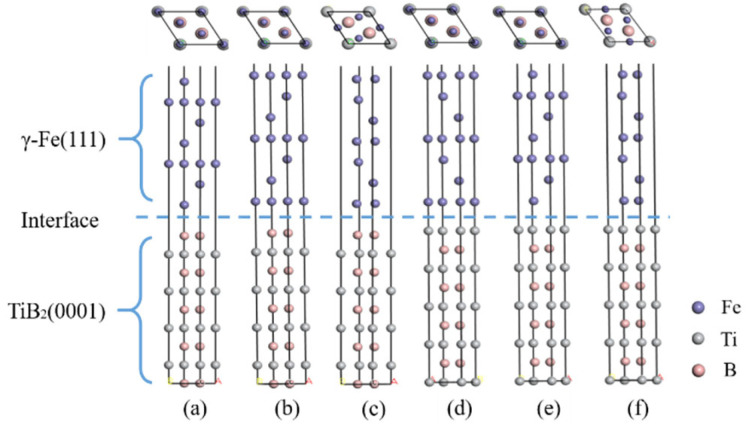
Six interface models of γ-Fe(111)/TiB_2_(0001): (**a**) B-top 1 site, (**b**) B-top 2site, (**c**) B-center site, (**d**) Ti-top 1 site, (**e**) Ti-top 2 site, (**f**) Ti- center site.

Based on the above calculation results, the interface structure of TiB_2_(0001)/γ-Fe(111) was formed by stacking 7-layers γ-Fe (111) and 9-layers TiB_2_(0001). In order to prevent the interaction between the upper and lower sides of the interface slab, a vacuum layer of 10 Å was constructed at the γ-Fe interface. The mismatch degree of the model is less than 15%, which meets the requirement of forming a semi-coherent interface. Six different interface models were established (B-top 1, B-top 2, B-center, Ti-top 1, Ti-top 2, Ti-center) in TiB_2_ (0001) and γ-Fe (111) as shown in Figure 3.

- Adhesion Work

The adhesion work (Wad) is a key parameter to characterize the interfacial bonding strength, which is a reversible work to separate the interface into two free surfaces [31]. The adhesion work (Wad) is determined as [32]:

(9) $$W_{ad}=\frac{1}{A}(E_{total}^{Fe}+E_{total}^{TiB2}-E_{total}^{Fe/TiB2})$$

where $E_{\mathrm{total}}^{{\mathrm{Fe}/\mathrm{TiB}}_{2}}$ is the total energy of fully relaxed interface, $E_{\mathrm{total}}^{\mathrm{Fe}}$ and $E_{\mathrm{total}}^{{\mathrm{TiB}}_{2}}$ denote the total energies of fully relaxed surface slabs, respectively. A represents the interface area.

Interfacial distance (d_0_) and adhesion work (W_ad_) after full relaxation are shown in Table 4. Besides that, the different termination and stacking sequences have a significant impact on the W_ad_ and d_0_. It can be seen that the B-terminated interfacial distance (d_0_) is significantly smaller than that of the Ti-terminated interface, but the B-terminated interfacial adhesion work (W_ad_) is larger. The larger the interfacial adhesion work and the smaller the interface distance, the stronger the interface stability is. The B-terminated “top 2” stacking structure exhibits the largest interfacial adhesion (4.16 J/m^2^) and the lower interfacial distance (1.24 Å), indicating that is the most stable and optimal. The B-terminated surface is more reactive and ready to form bonds.

- Interfacial Stability

The thermodynamic stability of the TiB_2_/γ-Fe interface can be further analyzed by calculating the interface energy. The interface energy (γ_int_) can be calculated as follows [33]:

(10) $$\gamma_{\mathrm{int}}=\frac{1}{A}[E_{total}-\frac{1}{2}N_{B}\mu_{TiB_{2}}^{bulk}+(\frac{1}{2}N_{B}-N_{Ti})\mu_{Ti}-N_{Fe}\mu_{Fe}^{bulk}]-\sigma_{TiB_{2}}-\sigma_{Fe}$$

where $E_{\mathrm{total}}$ is the total energy of TiB_2_/γ-Fe system; $\mu_{{\mathrm{TiB}}_{2}}^{\mathrm{bulk}}$ and $\mu_{\mathrm{Fe}}^{\mathrm{bulk}}$ stand for the chemical potential of the bulk TiB_2_ and Fe atoms, respectively. $N_{B}$ and $N_{\mathrm{Fe}}$ are the number of B and Fe atoms in the interface, respectively. $\sigma_{{\mathrm{TiB}}_{2}}$ and $\sigma_{\mathrm{Fe}}$ are the surface energies of the TiB_2_ and Fe surface structures, respectively. A is the interface area.

**Figure 4:**
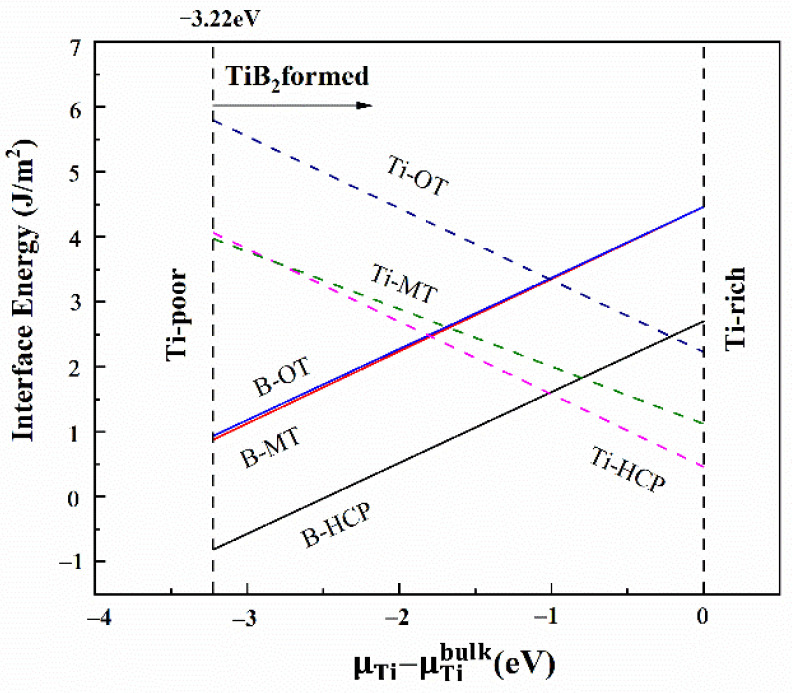
The relationship between TiB_2_ (0001)/γ-Fe(111) interfacial energy and different chemical potential of titanium.

Figure 4 shows the relationship between different chemical potentials of titanium and interface energy changes for six interface models. The interface energy of the B-terminated model increases with the increase of the chemical potential, while Ti-terminated model shows the opposite trend. Under the same termination, the interfacial energies of “top 2” model are the smallest. Obviously, the interface energies of B-top 2 and Ti-top 2 are −0.81~2.7 J/m^2^ and 4.06~0.46 J/m^2^, respectively, which are more favorable interface structures. In the subsequent analysis, we will focus on them.

- Electronic Structure and Bonding

The interfacial bonding strength and stability are closely related to the electronic structure and bonding of TiB_2_(0001)/γ-Fe(111) interface atoms. Electronic structure mapping is to determine the polarity of bonding according to the specific spatial distribution of different charge accumulation and depletion. The models used in this simulation are all ideal models which reach stable state after atomic relaxation, and the defects in crystal are not considered. The charge density difference and density of states of TiB_2_(0001)/γ-Fe(111) were analyzed in depth.

**Figure 5:**
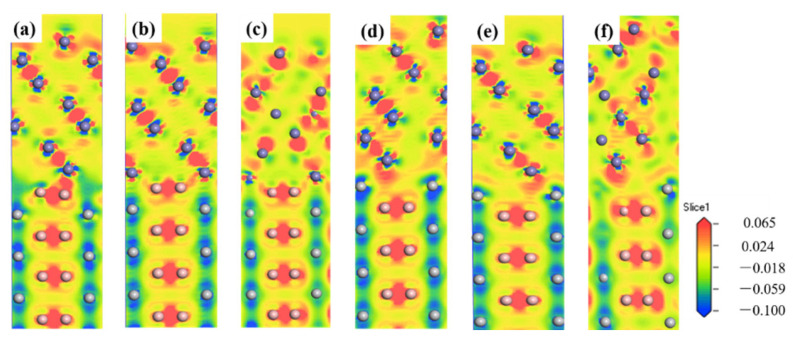
Charge density difference for the TiB_2_(0001)/γ-Fe(111) interfaces: (**a**) B-top 1; (**b**) B-top 2; (**c**) B-center; (**d**) Ti-top 1; (**e**) Ti-top 2; (**f**) Ti-center.

Figure 5 shows the charge density difference of six models. The blue and red regions represent the charge depletion and the charge accumulation regions, respectively. The B-terminated interface model has obvious charge localization characteristics, strong charge accumulation around B atoms, a small amount of charge accumulation and partial charge loss near Fe atoms, and almost all charge loss around Ti atoms. The strong electronegativities of the B atoms lead to electronic transfers from the Fe atoms to the interface, and most of them cross the interface and deposit near the B atoms, resulting in a strong Fe-B covalent bond. Some of the depleted Ti atoms combine with B atoms to form Ti-B covalent bonds, and some Ti atoms transfer to the interface and near the Fe atoms to form Fe-Ti metal bonds. The charge transfer in the “B-top 1” and “B-top 2” interface models are very obvious. Due to the stronger charge interaction between Fe-B atoms in the “B-top 2” structure, the interface space is smaller. The interaction between Fe and Ti atoms at the Ti-terminated produces metal bond, while the B atom is far away from the Fe atoms, only a weak covalent bond is produced, so the interfacial interaction is weaker. It is proved that the interface structure of “B-top 2” is more stable.

**Figure 6:**
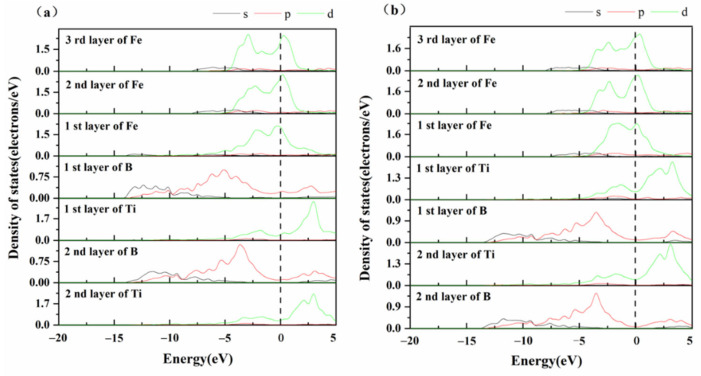
Partial density of states (PDOS): (**a**) B-top 2; (**b**) Ti-top 2.

Figure 6 shows the partial density of states (PDOS) of the interface structure of “B-top 2” and “Ti-top 2”. The interaction between atoms at the interface leads to the obvious difference between the PDOS curves of the interface atoms and those of the inner atoms. It also confirms that the charge distribution is localized. In the “B-top 2” interface model, the 3d orbital of the interface Fe atoms and the 3p orbital of the interface B atoms have obvious multiple resonance peaks between −7.5−2.5eV, the orbital hybridization occurred between two atomic orbitals at −13eV, which shows a strong Fe-B covalent bond. Compared with the internal Fe atoms, the interfacial Fe atoms have a wider energy density range at the Fermi level, and the Fermi level of the Fe atom is high, indicating that Fe-Fe metal bonds form. The interfacial B atoms have a higher density value at the Fermi level and show a higher filling state, indicating that there are obvious charge transfers between Fe atoms and B atoms. Figure 6b shows the density of states of the “Ti-top 2” structure. A pseudo-energy gap appears at the Fermi level of Ti atoms at the interface and the density value are even higher, showing that charge transfer and redistribution occurred between Fe and Ti atoms, resulting in strong Fe-Ti metal bonds. The density distribution of internal B atoms is similar, and there is only tiny orbital hybridization with the interface Fe atoms, forming the weaker Fe-B covalent bond. Due to the stronger effect of the Fe-B covalent bond, the “B-top 2” interface model is more stable.

#### 3.2.3. Tensile Strength and Property

- Tensile Simulation

**Figure 7:**
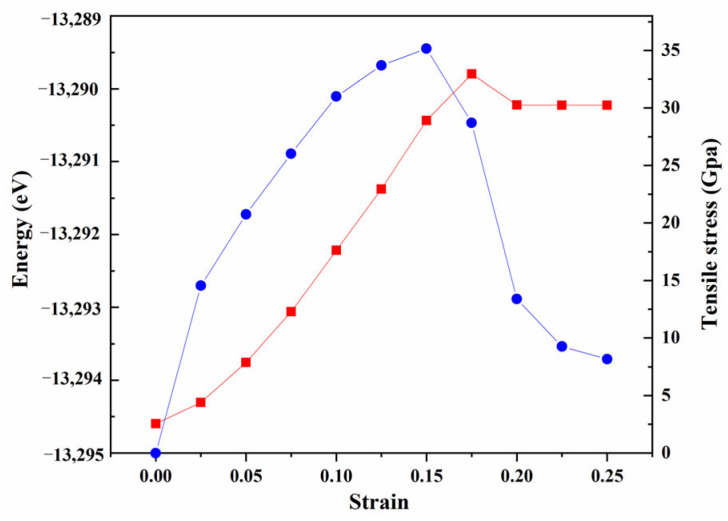
Profiles of tensile stress versus engineering strain (blue line) and deformation energy. (red line) of “B-top 2” interfacial model of TiB_2_(0001)/γ-Fe(111).

The uniaxial tensile test of the relaxed supercell was carried out by using the first principle to predict the tensile properties of the interface model theoretically. The specific method is to gradually increase the lattice length along the *z*-axis with 2.5% as the strain step length, and each new structural optimization is based on the previous optimization. As the strain increases, the atomic position of the whole system changes continuously until the fracture occurs. The normal strain is presented as follow:

(11) $$\sigma_{tensile}=(l-l_{0})/l_{0}$$

where l_0_ and l denote the original cell length and the stretched cell length, respectively. The red curve in Figure 7 shows the relationship between strain and deformation energy of the “B-top 2” interface structure. When the strain is less than 17.5%, the deformation energy increases linearly; Under the strain of 17.5%, the deformation energy reaches the highest point, and then decreases rapidly and is close to constant, indicating that the energy of the material tends to be basically stable and may fracture. The blue curve in Figure 7 shows the stress-strain curve of the “B-top 2” interface structure. The tensile process of “B-top 2” structure has gone through three stages. The first stage is the elastic deformation stage when the strain is less than 2.5%. Secondly, when the strain was between 2.5% and 15%, it was a typical plastic deformation stage. Finally, the material fractured when the strain exceeded 15%, but the stress did not drop sharply to zero, indicating that the “B-top 2” interface structure was the ductile fracture. The material completely breaks with the strain of 25%. Under the strain of 17.5%, the energy reaches the maximum, and the material has already broken. This is because the energy of the material does not change suddenly in the ductile fracture process. Even if the fracture occurs, some energies have not been completely released and remain in the material. When the strain increases further, the material is not enough to maintain the fracture energy generated in the fracture, the stress decreases sharply, and the energy also changes suddenly.

- Electronic property

**Figure 8:**
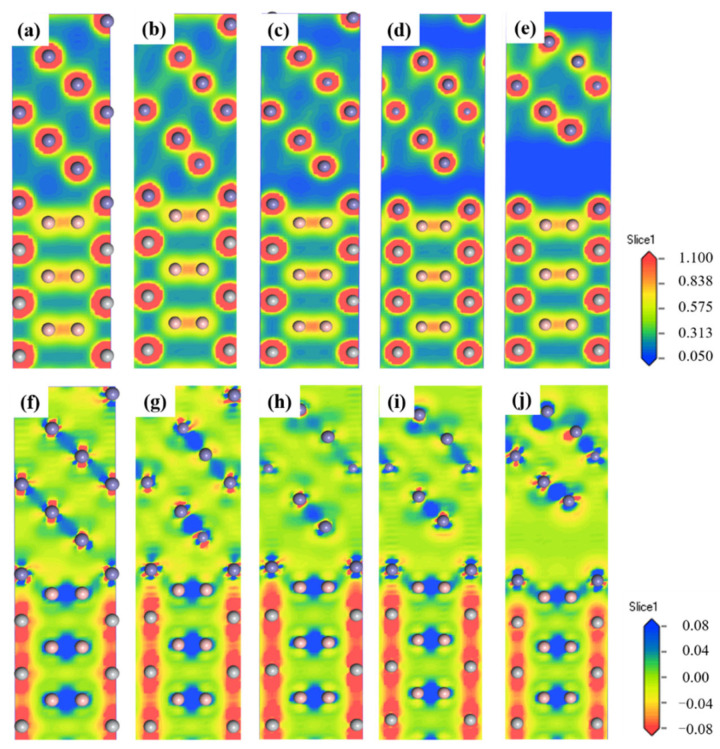
The charge density distributions (**a**–**e**) and charge density difference (**f**–**j**) of “B-top 2” interfacial model for TiB_2_(0001)/γ-Fe(111) at the strain (**a**,**f**) 0%; (**b**,**g**) 2.5%; (**c**,**h**) 15%; (**d**,**i**) 17.5%; (**e**,**j**) 25%.

According to the deformation energy and stress-strain diagram in Figure 7, the interface models with the strain of 0%, 2.5%, 15%, 17.5%, and 25% are selected to analyze the charge density distributions and charge density difference, as shown in Figure 8. Figure 8a,f are the charge distribution diagram at the strain of 0%. The Fe atom at the interface is closely combined with the B atom, which has localization characteristics, charge exchange and transfer occur, and the atomic arrangement is regular. The interface structure is stable and has no obvious change at the strain of 2.5%. When the strain is 15%, the distance between Fe atoms in the second layer and Fe atoms at the interface increases, the charge density begins to decrease, and the charge exchange with the interface density weakens. The obvious fracture occurs in the Fe atoms at strain 17.5%, and the Fe atoms are arranged disorderly, but the bonding at the interface is still tight. When the strain increases to 25%, the internal fracture occurs in the Fe condition and the charge density in the local region of the internal Fe atom increases. In the Fe atom, it is mainly composed of Fe-Fe metal bonds. In the TiB_2_ atom, there are Ti-Ti metal bond, Ti-B covalent bond and B-B covalent bond. Among them, Ti-Ti metal bond shows a charge density distribution similar to that of Fe-Fe metal bond. In addition, the charge density values of Ti-B and B-B covalent bonds are high, which is conducive to strengthening the internal stability of TiB_2_. There is a strong charge exchange between Fe and B atoms at the interface, indicating that the interfacial combination between Fe atom and B atom is stable. Because of the strong covalent bond of the Fe-B bond, the interface is not easy to fracture, and the Ti-B covalent bond in ceramic particles is also strong, however, the Fe-Fe bond is weak and the fracture is easy to occur in the Fe matrix. It is consistent with the previous conclusion. To sum up, TiB_2_ is beneficial to enhance the interfacial bonding strength of the Fe matrix.

In addition, the formation of Fe-B covalent bond is conducive to improving the bonding strength at the interface.

## 4. Analysis on TiB_2_ as Heterogeneous Nucleation of γ-Fe

The heterogeneous nucleation potential of ceramic particles on metal matrix is greatly affected by the interfacial energy between ceramic phase and metal [19,34]. When the interfacial energy between TiB_2_ and γ-Fe is lower than that between γ-Fe and Fe melt (0.24 J/m^2^), effective heterogeneous nucleation occurs [35]. The interfacial energy of the “B-top 2” model at the Ti-rich condition is −0.81 J/m^2^, which is significantly lower than that of the solid-liquid interface. The interfacial bonding strength is stronger and the stability is higher. Therefore, the “B-top 2” interface model is most conducive to the heterogeneous nucleation of TiB_2_ on γ-Fe. It is theoretically shown that TiB_2_ can be used as the heterogeneous nucleation substrate of γ-Fe.

## 5. Conclusions

TiB_2_/316L stainless steel composites were prepared by SLM. The adhesion work, interface energy, and electronic structure of the TiB_2_/γ-Fe interfaces were used to investigate the heterogeneous nucleation potential of γ-Fe grains on TiB_2_ particles using the first principle. The main conclusions are summarized as follows:

(1) Under the same terminated (B or Ti-terminated), the “top 2” interface model has higher adhesion work, smaller interface distance and interface energy. Under the same stacking sequence, the B-terminated interface model has higher adhesion work and lower interface energy than the Ti-terminated one. Therefore, the “B-top 2” interface model has a larger adhesion work and smaller interface energy, which is the most stable structure of the six interface models.

(2) By analyzing the fracture properties of the “B-top 2” interface structure, the bonding strength of the “B-top 2” interface is higher than that of the Fe matrix. Moreover, it is difficult to fracture at the interface and belongs to ductile fracture, so the interface bonding is more stable.

(3) The “B-top 2” interface is mainly composed of the strong Fe-B covalent bond, and the “Ti-top 2” interface mainly consists of Fe-Ti metal bonds. The strength of the covalent bond is obviously higher than that of the metal bond. Therefore, the interface of the “B-top 2” model is more stable.

(4) The interface energy of the “B-top 2” model at the Ti-rich condition is lower than that of γ-Fe/Fe melt, indicating that TiB_2_ particles can promote heterogeneous nucleation of 316L stainless steel.

## Figures and Tables

**Table 1:** Calculated lattice constants (a), volume (V0), bulk modulus (B), and formation enthalpy (ΔH) of bulk γ-Fe and TiB_2_.

Phase	Method	A (Å)	c (Å)	V_0_ (Å^3^)	B (GPa)	ΔrH (eV/atom)
γ-Fe	GGA_this work_	3.445	3.445	40.85	306	-
	GGA [24]	3.448	3.448	41.01	314.7	-
	Exp [25]	3.450	3.450	41.06	-	-
TiB_2_	GGA_this work_	3.029	3.228	25.655	231.53	−1.10
	GGA [26]	3.033	3.231	25.73	260.5	−1.04
	Exp [27]	3.03	3.229	25.67	-	-

**Table 2:** The convergence of the surface energy concerning slab thickness of γ-Fe(111).

Layer (N)	Surface Energy (J/m^2^)
	γ-Fe(111)
5	2.69
7	2.56
9	2.55
11	2.55

**Table 3:** The change of interlayer distances (%) of TiB_2_(0001) surfaces with different termination atoms after relaxation.

Surface	Termination	Interlayer	Thickness (N)				
			3	5	7	9	11
TiB_2_	Ti	Δ_1–2_	−5.48	−6.04	−5.56	−5.27	−6.49
		Δ_2–3_	-	−1.62	−3.33	−1.46	−2.27
		Δ_3–4_	-	-	−0.89	−2.84	−2.27
		Δ_4–5_	-	-	-	−1.05	−4.54
		Δ_5–6_		-	-	-	−0.34
	B	Δ_1–2_	−3.45	−6.45	−4.34	−4.26	−4.99
		Δ_2–3_	-	−2.60	−1.18	−1.50	−1.30
		Δ_3–4_	-	-	−0.39	−1.99	−1.91
		Δ_4–5_	-	-	-	−0.38	−1.70
		Δ_5–6_	-	-	-	-	−0.84

**Table 4:** Interfacial distance and adhesion work of TiB_2_(0001)/γ-Fe(111) after full relaxation.

Termination	Stacking Sequences	After Relaxation	
		d_0_ (Å)	W_ad_ (J/m^2^)
B-terminated	top 1	1.99	2.6
	top 2	1.24	4.16
	center	2	2.62
Ti-terminated	top 1	2.14	1.58
	top 2	2.11	3.2
	center	2.13	1.76

## Data Availability

All data reported in this paper is contained within the manuscript.
